# Molecular Epidemiologic Characterization of a Clustering HCV Infection Caused by Inappropriate Medical Care in Heyuan City of Guangdong, China

**DOI:** 10.1371/journal.pone.0082304

**Published:** 2013-12-03

**Authors:** Yi-Qun Kuang, Jin Yan, Yan Li, Xuhe Huang, Ye Wang, Guolong Yu, Xinge Yan, Ping Lin, Bing Qin, Peng Lin

**Affiliations:** 1 Guangdong Provincial Institute of Public Health, Guangdong Provincial Center for Disease Control and Prevention, Guangzhou, Guangdong, China; 2 Institute of HIV/AIDS Control and Prevention, Guangdong Provincial Center for Disease Control and Prevention, Guangzhou, Guangdong, China; 3 Guangdong Institute of Biological Products and Drugs, Guangdong Provincial Center for Disease Control and Prevention, Guangzhou, Guangdong, China; Institut Pasteur of Shanghai,Chinese Academy of Sciences, China

## Abstract

**Background:**

From November 2011 to January 2012, a number of clustering cases of HCV infection were reported in Zijin County, Heyuan City, Guangdong, China. Most patients in the clustering cases suspected that they could be infected due to inappropriate medical care in the clinic located at the Xiangshui road. However, the molecular epidemiology of the clustering cases remains unknown.

**Methodology:**

The residents, living at Xiangshui Road, with HCV antibody positive reported from 2011 and 2012 were recruited. A survey of the HCV infected individuals from the clustering cases was conducted. Each participant underwent a questionnaire defining demographic characteristics and health care history. HCV serological test and viral load test were performed to confirm the infection status. Molecular phylogenetic analysis and Bayesian coalescence analysis were conducted to further confirm the HCV subtype distribution and to reconstruct the associated demographic history and time-scaled phylogeny among the clustering cases.

**Principal Findings:**

The molecular phylogenetic analysis revealed that only two HCV subtypes, 2a and 6a, were found among the clustering cases. There was no close HCV subtype evolutionary relation was observed among patients from the same family. The 6a cluster showed higher viral loads than the 2a cluster. In addition, the Bayesian skyline plot analysis showed that both the HCV 2a and 6a subtype infections among the Heyuan cases experienced an “expansion-diminishment-expansion” featured dissemination. The 2a clustering infection occurred in 2004, and the 6a clustering cases originated in 2006.

**Conclusions:**

The molecular epidemiological characters imply that the inappropriate medical practices were possibly associated with the clustering HCV cases in Heyuan City during 2011, 2012. Latent HCV subtypes 2a and 6a infection may cause the prevalence and become a new public health issue in Guangdong, China.

## Introduction

Hepatitis C virus (HCV) is a main causative agent of chronic hepatic diseases, human hepatic cirrhosis and hepatocellular carcinoma [1]. HCV is an enveloped virus with a single-stranded positive sense RNA genome, which belongs to flaviviridae. HCV is divided into 6 genotypes and over 80 subtypes [2,3]. In China, 4 HCV genotypes (1–3,6) and related 13 subtypes are prevalent, which is becoming a severe public health burden to the government [4,5]. 

Hepatitis C widely distributes around the world, and people of different genders, ages, and races/ethnics are all susceptible to HCV. Transmission of HCV through parenteral exposures, blood or its products transfusion, occupational injury with a needle contaminated with blood, intravenous drug abuse, and tattooing has been well documented [6]. The most recent World Health Organization (WHO) estimate of the global prevalence of HCV infection is approximately 2.2-3.0%, with about 130-170 million HCV-positive people. The highest prevalence of HCV infection was found in the African and the Eastern Mediterranean region (> 10%). The areas characterized as a lower prevalence include Northern and Western Europe, Canada, and Australia (

< 1%) [7]. In China, whose population accounts for one-fifth of the global number, the overall prevalence of anti-HCV was 0.58%. For the age groups younger than ten, the anti-HCV prevalence was much lower than the older age groups [8]. In 2002, a survey of 12 counties of Guangdong showed that 2.25% people were HCV antibody positive [9]. A seroprevalence survey conducted by Guangzhou Blood Center showed that, among 559 890 donors, 1 877 (0.335%) were positive for anti-HCV. The anti-HCV rate was significantly higher for males than females, and significantly lower among the donors living in Guangdong Province than those who migrated from other locations. The seroprevalence of HCV among 559 890 first-time volunteer blood donors in China reflects a regional heterogeneity in HCV prevalence and changes in blood donor recruitment models [10]

. 

In recent years, the number of reported HCV cases has been increasing at an annual increasing rate of 23.3% in Guangdong (unpublished data). The reported cases are mainly distributed at Pearl River Delta and Western Guangdong where most of injecting drug users (IDUs) are located. From November 2011 to January 2012, clustering HCV infection cases were reported in Zijin County, Heyuan City of Guangdong province. Heyuan City, a relatively economy undeveloped city, is located in Northeast Guangdong. The total population of Zijin County was 812 159. The reported clustering HCV cases are mainly located on the Xiangshui Road, where was a joint part of three communities with a total population of 45 000. Most infected individuals suspected that they might be treated with an inappropriate medical care in a clinic on Xiangshui Road. To find out the origin of the clustering cases, a survey was conducted by reviewing the patients’ health care history records. Molecular phylogenetic analysis of the HCV genomic regions (Core and NS5B) and Bayesian Coalescent analysis based on the partial HCV Core-E1 region have been conducted to confirm the source of the exposure and to determine the origin of the HCV strains. 

## Materials and Methods

### Ethnic approval

This study was carried out according to the World Medical Association Declaration of Helsinki (http://www.wma.net/e/policy/b3.htm) and the Standard Laboratory Test Technology of HCV by the Chinese Center for Disease Control and Prevention. The procedures have been approved by the medical ethics committee of Guangdong Provincial Center of Disease Control and Prevention (GDCDC). All participants were voluntary in this study with written informed consents. Specifically, we obtained the written informed consents from the guardians on the behalf of children participants involved in the familial HCV infection analyses. 

### Study participants

A total of 184 HCV infected individuals living at Xiangshui Road, Zijin County, who were first diagnosed as HCV antibody positive in 2011 (33 individuals) or 2012 (119 individuals) were enrolled at the Center for Disease Control and Prevention of Zijin County, Heyuan City of Guangdong Province. Each HCV patient underwent a venipuncture firstly under agreement, and then an interview about their health care history. The subjects’ blood was collected and centrifuged, and the plasma was shipped to Guangzhou for HCV viral load testing, PCR amplification and sequencing for further analysis 172 subjects, 32 individuals in 2011 and 140 individuals in 2012, who were tested HCV antibody negative in the local hospital were enrolled in the study and used as the control.

### Serological testing and viral load measurement

HCV antibody status was determined by anti-HCV antibody testing ELISA Kit (Double Antigen Sandwich, Beijing WANTAI Biological Pharmacy). HCV positive samples were subsequently subjected to the viral load measurement using the HCV Fluorescent Polymerase Chain Reaction Diagnostic Kit (DAAN Gene). The samples with the viral load more than 1 000 copies/ml were determined as HCV RNA positive and proceeded to RNA extraction, RT-PCR amplification and sequencing. 

### Viral RNA purification, PCR amplification and sequencing

HCV genomic RNA was purified with 200 μl of plasma by the MagNA Pure LC RNA Isolation Kit (Roche) using the MagNA Pure LC instrument (Roche). Viral 1^st^ cDNA was synthesized from 7 μl extracted RNA with M-MLV reverse transcriptase under the manufacturer’s handbook (Invitrogen). Ten percent (2 μl) of the first-strand reaction was used for the first round of the nested Core or NS5B PCR. 

The primers for amplifying Core, NS5B, and E1 regions were described elsewhere [5]: For the Core region, the first round of PCR was conducted under the following conditions: 94°C for 5 min, then 30 cycles of 94°C for 30 sec, 55°C for 30 sec and 72°C for 1 min. The second round of PCR using nested primers was conducted under the same conditions except for annealing at 58°C for 30 sec. For the NS5B region, the first round of PCR was conducted under the following conditions: 94°C for 5 min, then 35 cycles of 94°C for 30 sec, 52°C for 30 sec and 72°C for 40 sec. The second round of PCR using nested primers was conducted with the same condition except for annealing at 52 °C for 30 sec. For the E1 region, the first round of PCR was conducted under the following conditions: 94°C for 5 min, then 35 cycles of 94°C for 30 sec, 50°C for 30 sec and 72°C for 1 min. The second round of PCR using nested primers was conducted under the same condition except for annealing at 52 °C for 30 sec and extending at 72°C for 40 sec. The inner primers were used to sequence the PCR product from both directions. For all amplified second round PCR products of three different HCV regions, the primers for the second round of PCR amplification were also used for sequencing from both directions. 

In addition, for samples with discrepancy in subtyping by Core and NS5B regions, we applied the extended Core-E2 and NS5B fragment PCR amplification to confirm the HCV subtype as previously described [11].

### Molecular phylogenetic analysis of HCV genotype

HCV genotype was determined by aligning to the reference sequences retrieved from the HCV database at Los Alamos (http://hcv.lanl.gov/content/sequence/NEWALIGN/align.html) followed by phylogenetic analysis [12]. The reference sequences were selected from all 7 genotypes to make sure all subtypes are included for accurate subtyping of the samples. For the reference subtypes containing more than two sequences, we chose one sequence from one country or region for each subtype. Sequences were edited by Sequencher 5.0 (Gene Codes Corporation) and aligned in Clustal X 2.1 (http://www.clustal.org/). The Phylogenetic analysis was performed with MEGA 5.2 software. The Neighbor-Joining (NJ) method was used with 1 000 bootstrap replications under Kimura 2-parameter substitution model with transitions and transversions (5,11). 

### Bayesian coalescent analysis of HCV subtypes

The Bayesian coalescent analysis was performed using the Bayesian Evolutionary Analysis Sampling Trees program (BEAST, version 1.7, http://evolve.zoo.ox.ac.uk/Beast/). This program applies an algorithm of Markov-Chain-Monte-Carlo (MCMC) chain to estimate the Bayesian inference. The 2a and 6a E1 reference sequences were collected from the HCV database at Los Alamos and GenBank. Only the sequences with pertinent sampling date and country/region information were downloaded from the HCV database at Los Alamos. For GenBank sequences, the blasted sequences with high score, exact sampling date and country/region records were chosen. The E1 region sequences of HCV 2a cluster or 6a cluster with references were aligned using the Clustal X 2.1. Prior to the Bayesian coalescence analysis of 2a or 6a dataset, model test was performed to choose the best substitution model for each subtype in jModelTest 2.1.3 [13,14], the ACI, AICc and BIC criteria were tested for both clusters. For the 2a dataset, the HKY+I+G model was found to be the best under all three criteria. As to the 6a dataset, the AIC and AICc favors GTR+I+G and K80+I+G model respectively, while the HKY+I+G was shown to be the best model based on the BIC criterion. We selected the HKY+I+G model for the 6a dataset. Furthermore, we combined the HKY+I+G model with the Strict clock, uncorrelated Lognormal relaxed clock or uncorrelated Exponential relaxed clock and Bayesian skyline model as previously suggested [15-18]. MCMC-chain length was set to 100 000 000 or 200 000 000 repeats to reach a sufficient effective sample size (ESS) of over 200 (at least over 150, which is higher than the acceptable level 100) for the analysis of 2a dataset or 6a dataset respectively. The BEAST results were further analyzed and demonstrated using the MCMC Trace File Analyzer program Tracer (version 1.5, http://tree.bio.ed.ac.uk/software/tracer/). The ‘‘Bayesian Skyline Reconstruction’’ method was used to reconstruct the demographic history of HCV infections with an assumption that the viral transmission parameters remain constant through time under the best model combination tested. 

Next, the TreeAnnotator program (version 1.7, http://evolve.zoo.ox.ac.uk/Beast/) was used to construct the phylogeny, which best summarizes the set of credible trees and is called the maximum clade credibility (MCC) tree. After discarding 10% of burn-in, a time-scaled phylogeny was estimated from a posterior distribution of trees that were generated under a given model combination. The generated phylogeographic structure trees were then visualized and decorated in the FigTree program (version 1.4, http://tree.bio.ed.ac.uk/software/figtree/). 

### Nucleotide sequence accession numbers

The nucleotide sequences of HCV Core and NS5B partial regions determined in this study have been deposited in the GenBank sequence database, and the accession numbers are as follow: JX194813-JX195062. 

## Results

### Demographic characteristics

A total of 184 residents previously reported as HCV antibody positive were recruited. The major demographic characteristics based on the questionnaires of the infected individuals were listed in Table *1*. Our results showed that the gender was distributed evenly in the case groups. The main age group of the infected cases was of age older than 40. The main occupations were housewives or unemployment, students, and peasants. The main education levels were primary school and secondary school. 

**Table 1:** Major demographic characteristics of 184 HCV-infected cases and 172 controls.

		Case		Control	
		No.	%	No.	%
Gender	Male	75	40.8	82	47.7
	Female	109	59.2	90	52.3
Age	<15	51	27.7	60	34.9
	15-39	32	17.4	52	18.6
	40-	101	54.9	60	58.7
Occupation	Students	47	26.1	66	40
	sporadically children	3	1.7	4	2.4
	Kindergarten children	7	3.9	12	7.3
	Peasants	28	15.6	12	7.3
	Housewives/unemployed	61	33.9	24	14.5
	Employed	2	1.1	17	10.3
	Teachers	3	1.7	5	3
	Retired	9	5	10	6.1
	Others	20	11.1	15	9.1
Education	Kindergarten	13	7.1	21	12.4
	Primary School	70	38.3	31	18.2
	Secondry School	58	31.7	49	28.8
	High School	22	12	42	24.7
	Graduate	4	2.2	25	14.7
	Illiterate	16	8.7	2	1.2

### HCV antibody and viral load testing

HCV antibody ELISA was performed to check the recruited patients’ HCV antibody status. Our serological study demonstrated that all the (184/184) blood samples were HCV antibodies positive, which suggested that the HCV infection occurred in them. In the patients with chronic hepatitis C, the HCV RNA level is an important predictor of treatment response and prognosis. Next, the HCV viral genomic RNA levels were measured with the patients’ plasma. 

The plasma viral loads of 184 HCV antibody positive blood samples were tested, in which 132 (71.7%) were determined as HCV RNA positive (HCV RNA > 1 000 copies/ml). In these samples, 8 samples have a viral RNA level higher than 10^3^ copies/ml, 22 samples higher than 10^4^ copies/ml, 33 samples higher than 10^5^ copies/ml, 57 samples higher than 10^6^ copies/ml, and 12 samples higher than 10^7^ copies/ml. 

### HCV subtype distribution among infected individuals

For the 132 HCV RNA positive patients, the partial sequence of Core and NS5B genes was amplified and sequenced respectively. The Core sequences of all samples (132, 100%) were successfully obtained. 123 (93.2%) NS5B sequences were successfully obtained but failed in 9 (6.8%) samples. 

**Figure 1:**
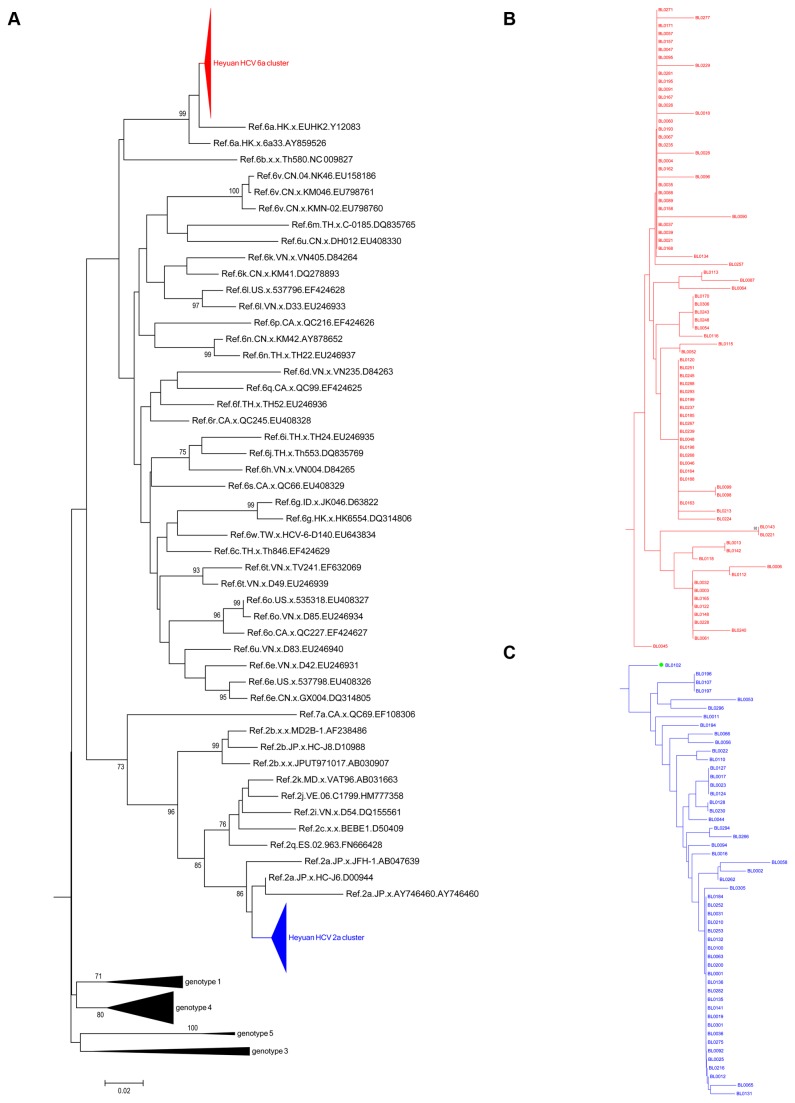
HCV molecular phylogenetic tree based on the Core sequences. The Neighbor-joining phylogenetic tree was constructed based on the Core gene containing 374 nucleotides, corresponding to 332-705 of H77 genome (NC_004102) (A). The red filled triangle indicates the 6a cluster of Heyuan HCV-infected cases; the blue filled triangle indicates the 2a cluster. Genotypes 1, 3, 4, and 5 were collapsed into black filled triangle. The subtrees of Heyuan HCV 6a cluster (B) and 2a cluster (C) display the detailed phylogenetic relations of all Heyuan HCV cases. Bootstrap values are based on 1 000 replicates and statistic values > 70% are indicated at the nodes of the corresponding branches. The scale bar unit is substitutions/site.

**Figure 2:**
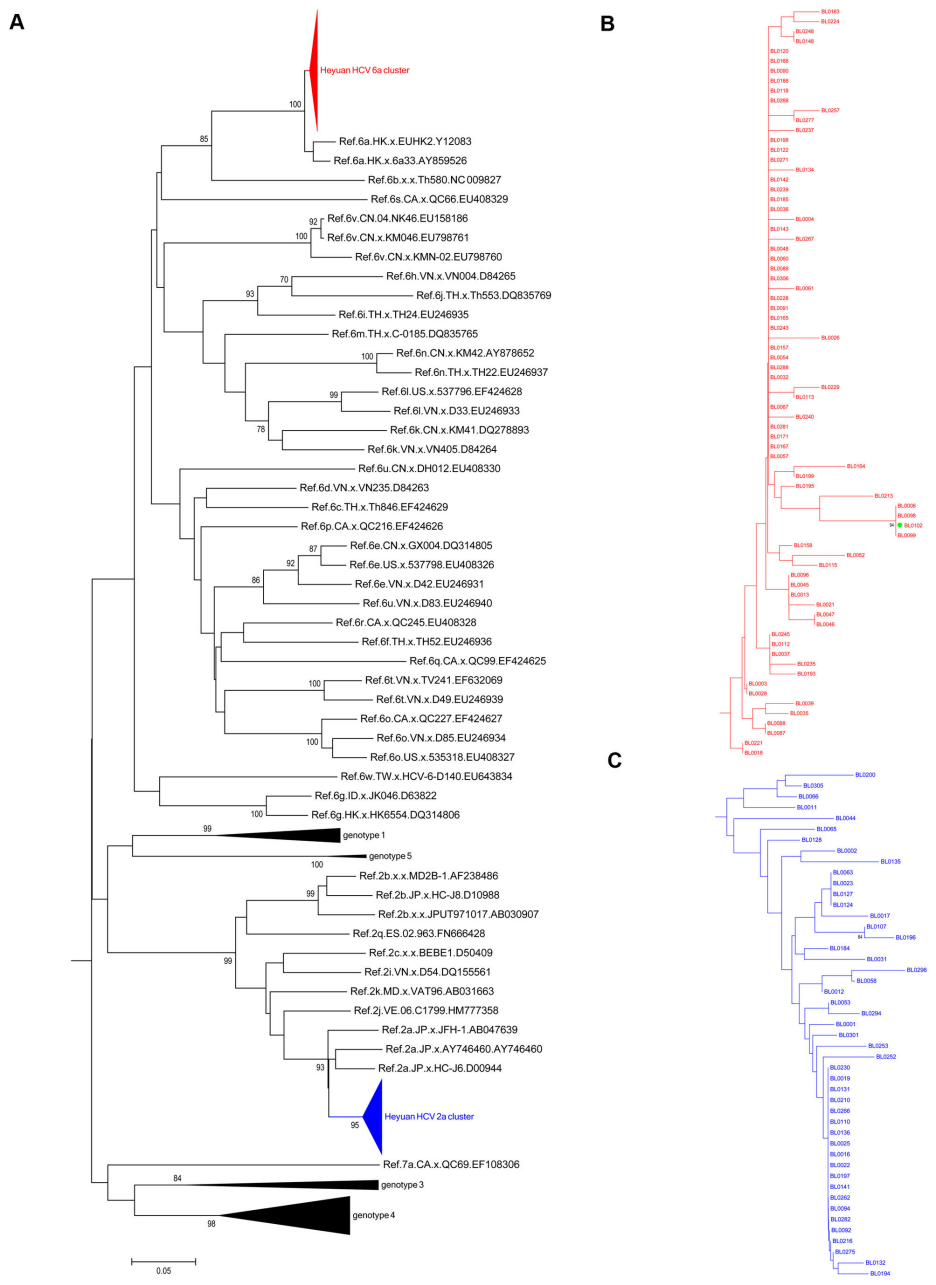
HCV molecular phylogenetic tree based on the NS5B sequences. The Neighbor-joining phylogenetic tree was constructed based on the NS5B gene containing 354 nucleotides, corresponding to 8268-8621 of H77 genome (NC_004102) (A). The red filled triangle indicates the 6a cluster of Heyuan HCV-infected cases; the blue filled triangle indicates the 2a cluster. Genotypes 1, 3, 4, and 5 were collapsed into black filled triangle. The subtrees of Heyuan HCV 6a cluster (B) and 2a cluster (C) display the detailed phylogenetic relations of all Heyuan HCV cases. Bootstrap values are based on 1 000 replicates and statistic values > 70% are indicated at the nodes of the corresponding branches. The scale bar unit is substitutions/site.

With the obtained HCV sequences in patients and appropriate reference sequences from HCV genotypes and subtypes, two phylogenetic trees were constructed based on the Core and NS5B regions. The overall topology of the two phylogenetic trees is similar (Figure 1 and Figure 2), and most isolates are generally located in the same position in the two different trees. In the Core-based phylogenetic tree, 81 isolates from Heyuan HCV cases (indicated in red triangle and designated as Heyuan HCV 6a cluster) are all clustered with the subtype 6a reference variants, and closely related to two Hong Kong-derived strains (accession numbers Y12083 and AY859526) with a bootstrap value of 99% (Figure 1A). In the other hand, the other 51 Heyuan HCV cases are clustered with the 2a subtypes (indicated in blue triangle and designated as Heyuan HCV 2a cluster, Figure 1A). The specific phylogenetic clades of Heyuan HCV 6a cluster and 2a cluster were expanded in Figure 1B and Figure 1C. However, in the phylogenetic tree based on the NS5B region among 123 Heyuan HCV cases, 76 isolates are clustered with subtype 6a (red triangle, Figure 2A) and the left 47 cases are clustered with subtype 2a (blue triangle, Figure 2A). These two phylogenetic clades were demonstrated in more details in Figure 2B and Figure 2C respectively. 

**Figure 3:**
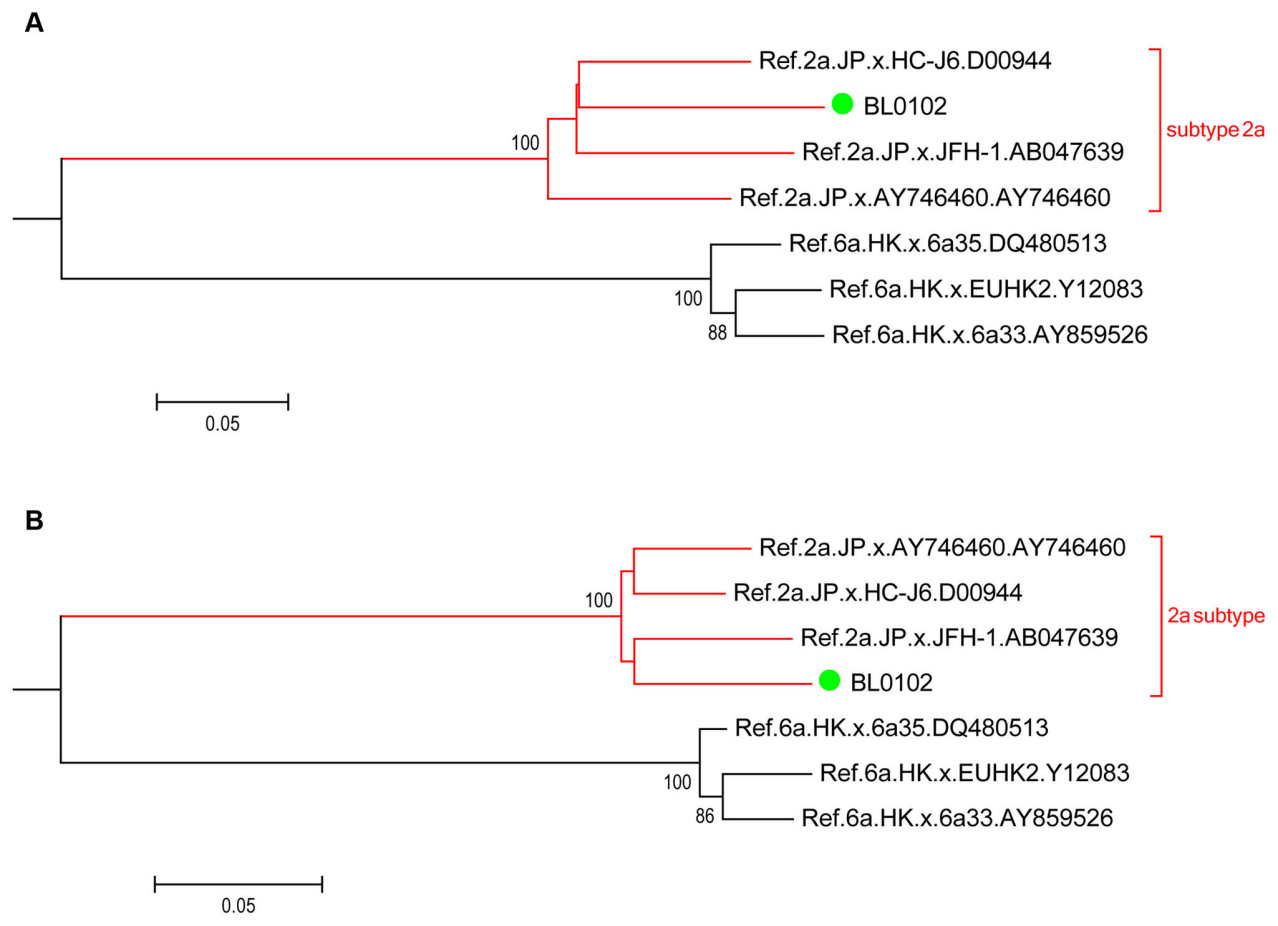
Subtype analysis of Heyuan HCV case BL0102. The Neighbor-joining phylogenetic trees were constructed based on the extended Core-E2 (A) and the NS5B (B) near full length fragments respectively. The BL0102 case was highlighted in green filled circle, subtype 2a branch was bracketed in red square. Bootstrap values are based on 1 000 replicates and statistic values > 70% are indicated at the nodes of the corresponding branches. The scale bar unit is substitutions/site.

Interestingly, a discrepancy of subtyping was observed in sample BL0102 when using the partial region fragments of the Core and the NS5B genes (indicated in green filled circle in Figure 1C and Figure 2B). Thus, the longer fragments encompassing Core-E2 and NS5B near full length were amplified respectively in BL0102. The phylogenetic trees constructed based on the Core-E2 fragment (Figure 3A) and the NS5B near full length fragment (Figure 3B) revealed that BL0102 is a HCV subtype 2a isolate. 

### HCV phylogeny among family members, correlationship analysis between viral loads and HCV subtypes

**Figure 4:**
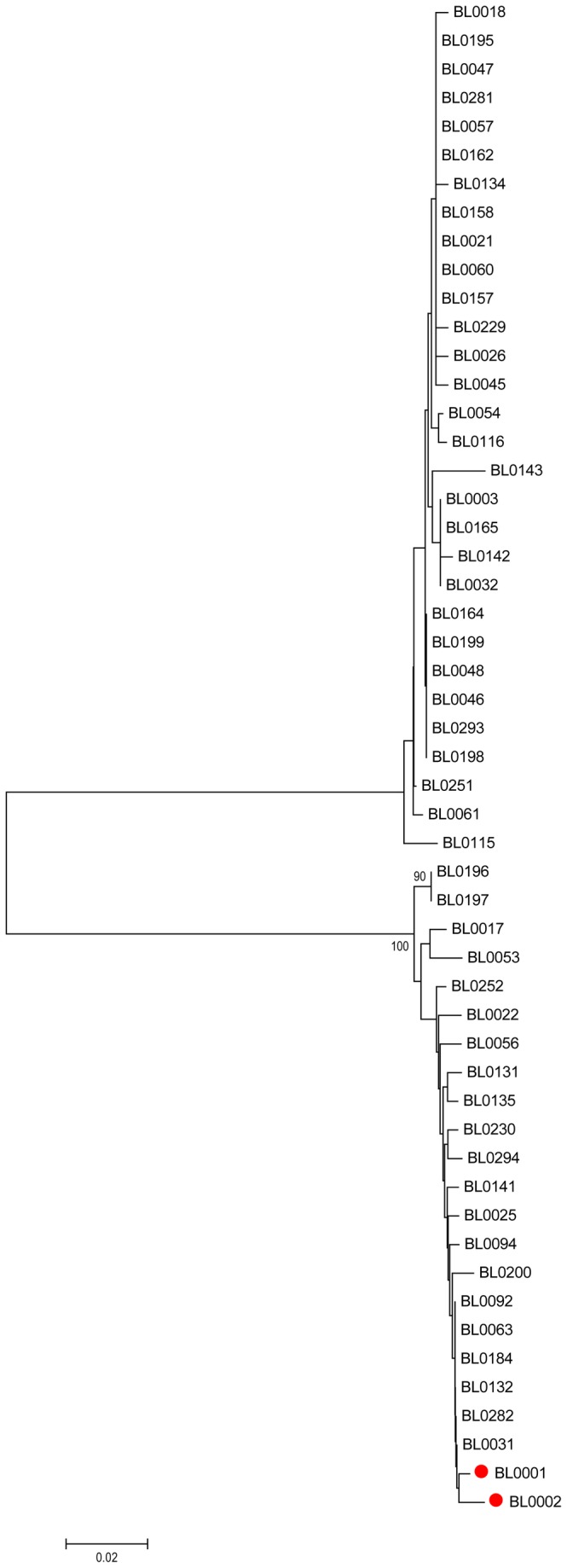
Phylogenetic tree analysis among family members. The Neighbor-joining phylogenetic tree was constructed based on the HCV Core gene from 53 cases in 23 families. The only cases from the same family that are clustered together (BL0001 and BL0002) were highlighted in red filled circle. Bootstrap values are based on 1 000 replicates and statistic values > 70% are indicated at the nodes of the corresponding branches. The scale bar unit is substitutions/site.

Among the 132 HCV cases, there were 53 patients that belonging to 23 families with more than one patient, of which 4 families were parents/child, 1 family was father/child, 7 families were mother/child, 4 families were spouses, 5 families were grandparent/child, and 2 families were siblings. Based on the HCV Core sequences, we constructed a phylogenetic tree for the cases of the 23 families. The results demonstrated that there was no obvious clustering of cases among each family, except for the BL0001 and BL0002 (red filled circles, Figure 4). 

**Figure 5:**
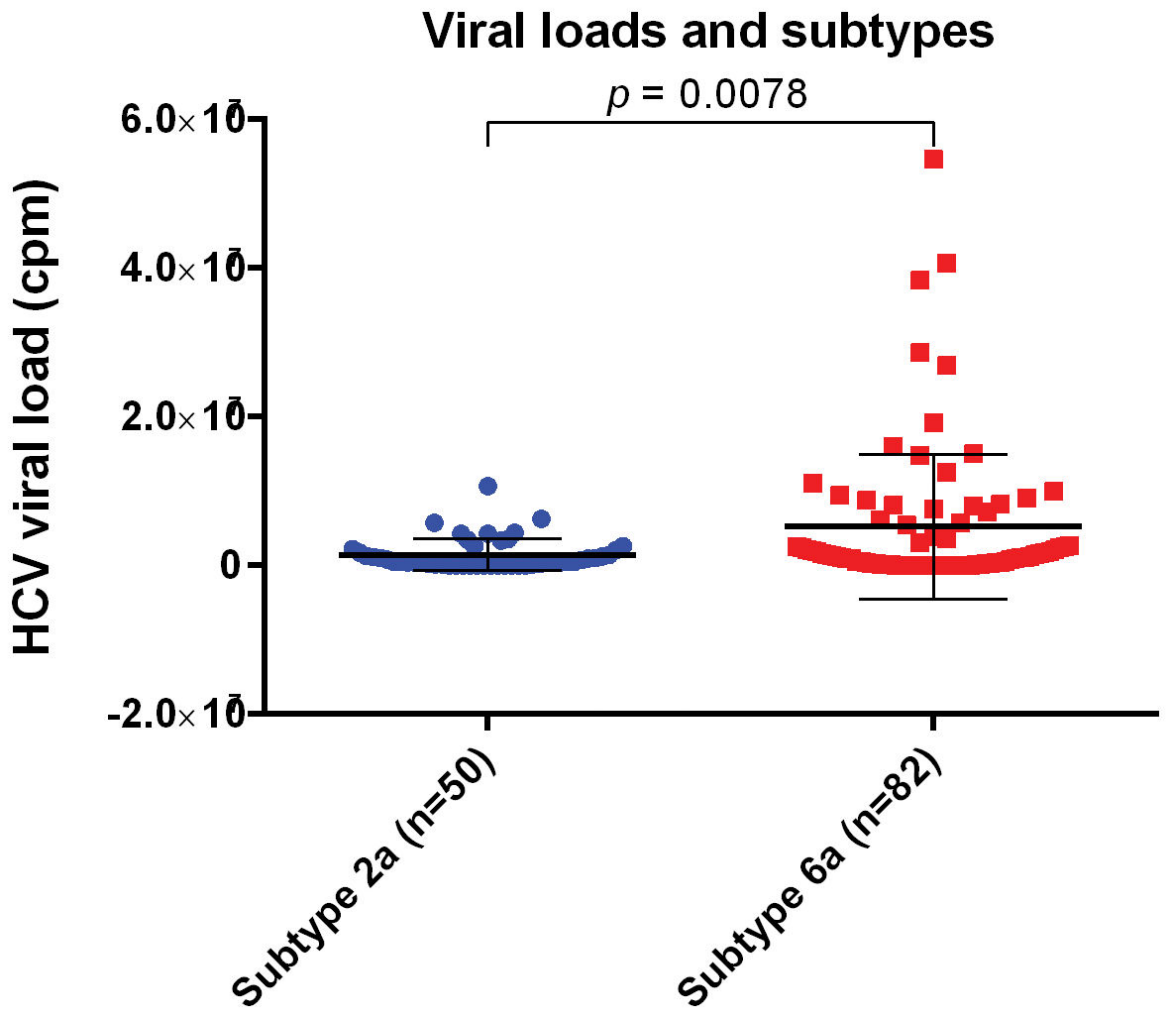
HCV viral loads and subtypes. Plasma viral loads of 50 HCV 2a cluster cases and 82 HCV 6a cluster cases were compared. Statistical analysis was performed by two-tailed *p* value in GraphPad, unpaired *t* test result *p* < 0.01 was considered significant.

Next, based on the accurate subtyping of the 132 Heyuan HCV cases, we sought to see the association of the viral loads with the subtypes. The HCV viral loads between 2a cluster and 6a cluster were compared. The results showed that the viral loads of the 6a cluster were significantly higher than those of the 2a cluster (*p* = 0.0078) (Figure 5). 

### Spreading history of HCV 2a and 6a cluster infections

**Figure 6:**
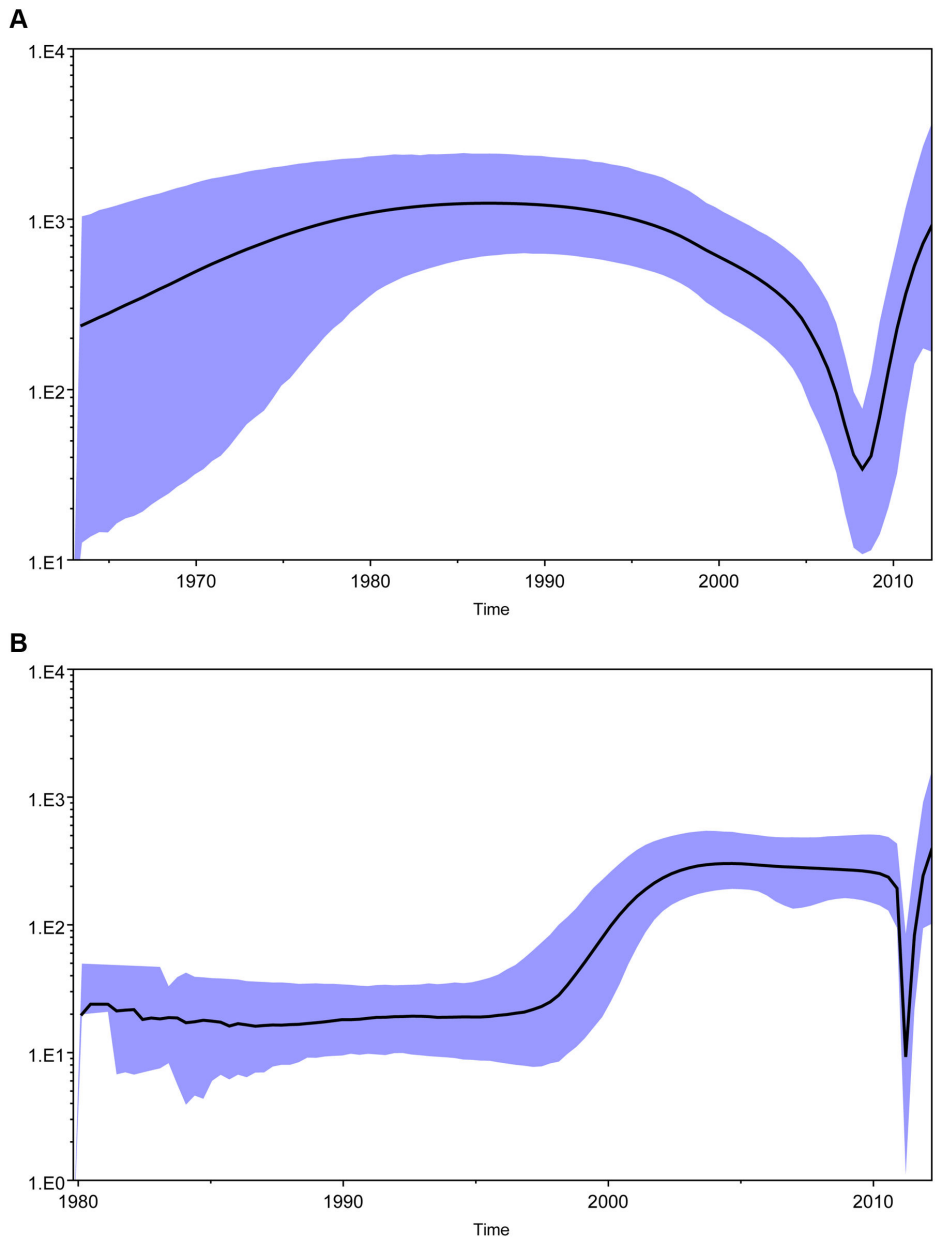
The reconstructed Bayesian skyline plot of the history of Heyuan HCV 2a and 6a clusters. Bayesian skyline plot was reconstructed to estimate the effective infection numbers of Heyuan 2a cluster (A) and 6a cluster (B). X-axis is the year of the infection, Y-axis indicates the estimated HCV infection population size. Black line represents the estimated effective population size through time. The blue area represents the 95% upper and lower highest posterior density (HPD) confidence intervals for this estimate.

According to the accurate classification of HCV subtypes 2a and 6a among Heyuan HCV cases, we amplified the E1 region for the molecular clock analysis of each cluster. The Bayes Factor (BF) in Tracer demonstrated that the HKY+I+G model combined with the uncorrelated Lognormal relaxed clock model for Bayesian skyline plot (BSP) analysis is the best-fitting model for both 2a cluster (Ln BF = 77) and 6a cluster (Ln BF = 133). The Bayesian skyline plots were reconstructed for both the 2a cluster and the 6a cluster based on the best model combinations. The result demonstrated that both the 2a and 6a clusters experienced an “expansion-diminishment-expansion” overall spreading feature, although distinct transmission existed in between (Figure 6). The data showed that the 2a cluster has a steady growth in the number of effective infections between 1960 and 1989, and decreased relatively fast until reaching the lowest level in 2008. After that a sharp expansion persisted until the day of the outbreak (Figure 6A). However, the 6a cluster transition underwent a relatively steady level from 1980 to 1995, and then expanded at a logarithmic growth rate until reached a plateau in 2005. Then, after a stable period, the number of effective infections decreased sharply in 2011, and rebound fast soon until the outbreak day (Figure 6B). 

### Estimated Year of origin for HCV 2a and 6a clusters

**Figure 7:**
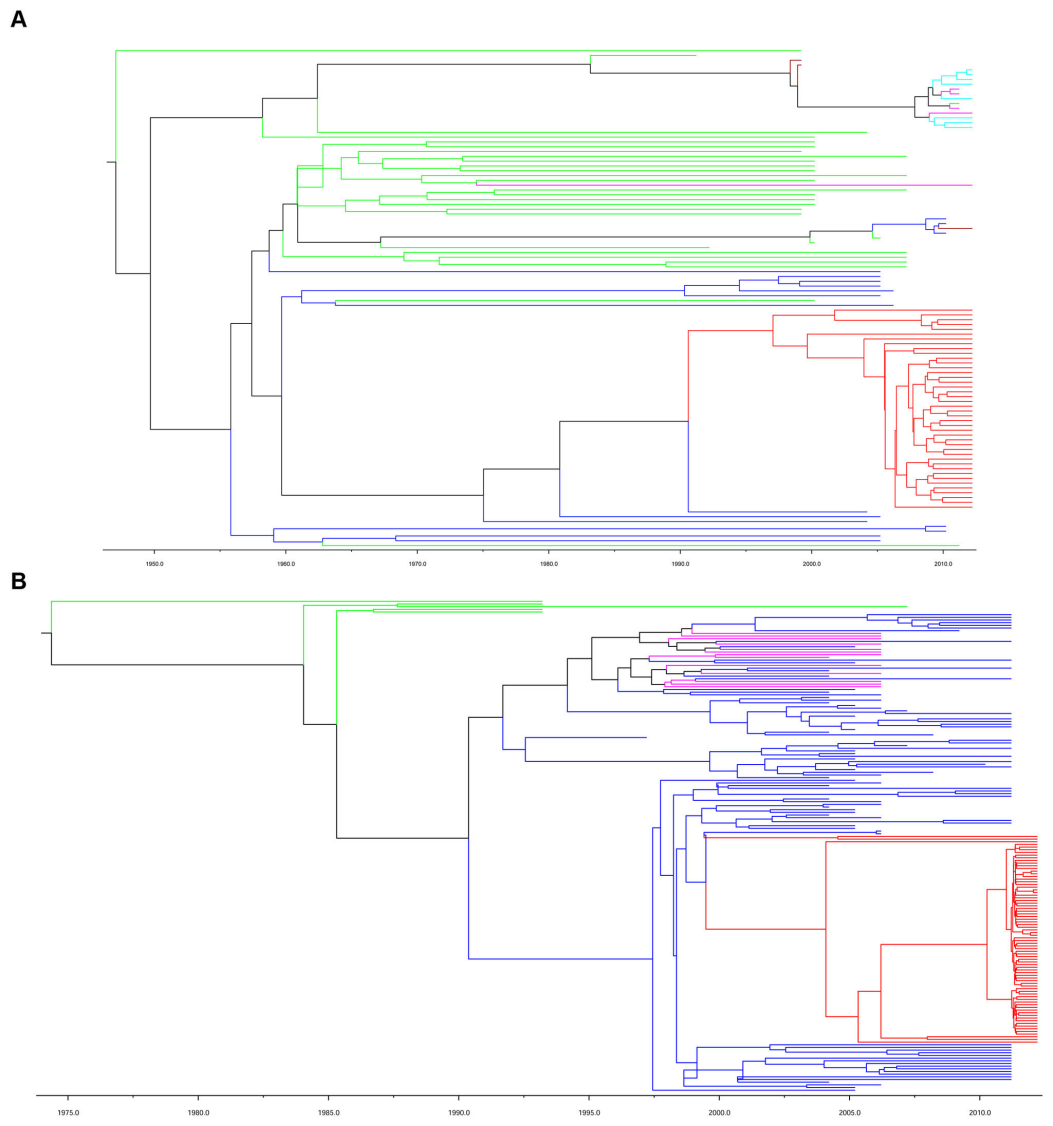
MCC trees of 2a and 6a clusters estimated under the model of Bayesian skyline plot model combinations. The MCC phylogenetic trees for Heyuan 2a cluster (A) and 6a cluster (B) were constructed under the best model combination tested. Branches are colored according to their respective geographic origins: (A). Heyuan 2a cluster, red; China, blue; Japan, green; United States, brown; Spain, Cyan; Denmark, magenta. (B). Heyuan 6a cluster, red; China, blue; Hong Kong, magenta; Vietnam, green. Scaled line indicates the different corresponding years the branches originated.

The MCC trees of HCV genotypes 2a and 6a were reconstructed under the best model combination with the E1 sequence datasets. The overall topology of the MCC tree agreed closely with that of the NJ tree in the 2a and 6a clusters (Figure 1, 2, 7). In the 2a MCC tree, three major clades, the Chinese clade (blue lines), the Japanese clade (green lines) and the European-American clade (magenta, brown and cyan lines) are clearly distinguished (Figure 7A). The Heyuan HCV 2a cluster (red lines) is enclosed in the Chinese clade, diverged with a Guangzhou strain from China (accession No. GQ206062) in 1988. Three major clades were observed in the Heyuan 2a cluster, and a large group originated in 2002 which accounts for most cases (Figure 7A). 

In the 6a MCC tree, there were two conspicuous major clades, representing Vietnamese clade (green lines) and Chinese clade (blue lines) respectively. The strains of Hong Kong (magenta lines) and the Heyuan 6a cluster (red lines) were contained in the Chinese clade (Figure 7B). The Heyuan 6a cluster diverged with two Guangzhou strains (accession No. GQ205860 and GQ205863) in 1999, and the major Heyuan cases originated in 2010. 

## Discussion

In China, the two predominant HCV subtypes, 1b and 2a, are generally caused by poor social and medical conditions, as well as the medical malpractice, with distinctive transmission and evolution features [19-21]. One cluster of the 1b subtype originated in 1968 and dispersed fast between 1968 and 1990. In particular, a large number of non-professional or poor trained healthcare workers entered the healthcare system during the Cultural Revolution from 1966 to 1976, and medical malpractice has become the main reason for the HCV spread. The other 1b cluster which originated in 1975 and spread rapidly during 1975 and 1985, was mainly due to the use of contaminated blood and blood products [22-24]. The HCV subtype 2a underwent the similar history in China. The HCV prevalence caused by medical exposure had happened in the last 50 years, as Fu and colleagues reported [10]. In the study, statistical analysis of factors associated with Heyuan HCV clustering infection showed that intravenous infusion, endoscope exposure, and partner/mother/children HCV infection were possibly associated with this case (Table *S1*). However, there were no cases of proven transmission of HCV when the endoscopes were reprocessed by using currently accepted standards [25]. Although the recommended decontamination procedures do not entirely eliminate the persistence of a small number of organisms on a few endoscopes, they are unlikely to cause blood borne viral infection transmission [26]. Unfortunately, the improper endoscope maintenances are still common in some hospitals in China. A survey of 122 endoscopy units found that, the disinfectant time shorter than 45 min and reusing of disposable materials still exist. Improvement is needed in the practice of endoscopy reprocessing, especially in middle-sized and small cities [27]. Since the clinic on Xiangshui Road did not provide endoscope service, the relationship between HCV infection and taking endoscope in the clinic was not analyzed. Although partner/parents/children HCV infection was an associated factor, no HCV molecular evolution relation was found between family members in our study (Figure 4). The limitation in the epidemiology aspect of this study is that the records in the clinic on Xiangshui Road were incomplete. Therefore, we could not confirm the healthcare history of some patients who claimed that they had taken treatment in this clinic, thus the bias of the analyses could not be corrected with these records. It calls for more sufficient epidemiological evidence to prove that the improper medical practice is the direct reason which caused the Heyuan HCV clustering infections.

In our study, the molecular phylogenetic analysis based on HCV Core and NS5B showed that there were only two HCV subtypes, 6a and 2a, in these clustering cases (Figure 1 and 2). This further proves the common origin of the clustering cases, probably in the clinic on the Xiangshui road as suggested by the questionnaires and the epidemiology statistics analysis (Table S1). The interspousal HCV infection including sexual and non-sexual transmission in acute and chronic hepatitis C patients is controversial [28]. Sexual transmission of HCV is generally considered to be uncommon [29]. But research in Japan also found that interspousal infection might be one of the important sources of acute HCV infection [30]. The intrafamilial, non-sexual household transmission of HCV had also been reported [31,32]. In our study, regarding 23 families with more than one patient, though these family members were clustered into two groups with other infectors, no family members showed a close evolution (Figure 4). Therefore, it strongly implied that they acquired HCV infection independently of each other. In addition, interestingly, one sample (BL0102) that was classified into different subtypes (6a or 2a) when analyzed based on the different HCV regions (Core or NS5B) was finally confirmed to be of the 2a subtype (Figure 3), suggesting that there was no mixing infection by multiple subtypes or recombination occurred among the Heyuan HCV clustering cases [15]. 

China is one of the most affected regions of HCV infection. Among the currently prevalent HCV genotypes 1, 2, 3 and 6, the subtype 1b prevails in the South, and the subtypes 1b and 2a prevail in the North [4]. In recent years, the 6 and 3 genotypes are transmitting fast in the southwest and becoming the predominant epidemic genotypes, which shows that the HCV genotypes in injection drug users are transmitting to the general population [24,33]. Fu and colleagues speculated that the 6a subtype dispersing in Guangdong is originated from the Vietnam strain in 1970s, when a large number of refugees from Vietnam immigrated into Guangdong during the late 1970s and early 1980s [15,23], while Hong’s study showed that the rapid spread of HCV in Southeast China was caused by the intravenous drug users from 1997 to 2000 [34]. Our data is consistent with previous reports (15,23,34) and also demonstrated the origin of the Heyuan HCV cases from Vietnam (Figure 7B), which implies the 6a subtype in Guangdong has become the dominant genotype [22]. Moreover, our data displayed a high percentage of HCV 6a infection in the clustering cases, and the 6a cluster has higher plasma viral loads than the 2a cluster (Figure 5), which was consistent with the fact that the 6a subtype is spreading at a high increasing trend among high-risk people [15,35]. Guangdong, the neighboring province of Guangxi, is in the drug transportation routes in China. Estimated epidemic in 2011 of Zijin County showed that the drug users were about 0.2% of the population. In the meantime, the sentinel surveillance in Zijin County of Heyuan City in 2011 showed that HCV infection in drug users was about 65% [36]. The existence of drug users in these clustering cases could not be excluded. However, it was a limitation that drug using investigation was not included in our questionnaire. 

HCV 2a is the other major subtype (38%) in the clustering infections in this study. Study on the first-time volunteer blood donors demonstrated that the prevalent HCV subtype 2a strains can be divided into two distinct clusters [23]. The Heyuan HCV 2a cases appear to be the first cluster that is domestically distributed (Figure 7A). In recent years, the proportion of type 2a has suffered a marked downward trend [23,24], resulting from an improved medical condition and high sustained virological response rates in this genotype [37]. However, previous study suggests that Guangdong acts as an initial origin of Zhenjiang 2a strains [38]. The HCV 2a prevailed in the Heyuan cases indicates that the 2a is a potential dissemination subtype, even though it has been replaced by the 6a and becoming less prevalent in Guangdong [23]. 

The analyses of the BSPs of Heyuan HCV 2a and 6a clusters depicted a distinct “expansion-diminishment-expansion” pattern representing the HCV infection population in both clusters (Figure 6). The 6a cluster underwent a rapid HCV population growth from 1995 to 2004, which was also similarly observed in a recent study including HCV subtypes 1b, 2a, 3a, 3b, and 6a prevailing in Southern China [39]. In addition, there was a considerable feature of the two BSP patterns that an abrupt drop in the HCV infected population growth in one to two years before or after the year 2010. According to the official reports, the first HCV case of the Zijin County of Heyuan City was identified in 2006, and the first HCV case in the town of the county where the Xiangshui Road locates and the clustering HCV cases happened in 2008 (unpublished data). Therefore, the abrupt drop of infection population growth of both 2a and 6a clusters in 2010 before and after is possibly attributed to the awareness and the control of HCV transmitting routes by the local government. However, the latent HCV infected patients developed hepatitis symptoms and the clustering cases eventually unmasked in 2011.

For the patients with liver disease, especially the elder patients, 1b or 2a is still the main prevalent subtype [40]. Our work of the molecular epidemiology of Heyuan HCV cases indicates that the 2a subtype prevailed in the clustering population, this will benefit to the therapy strategy and prognosis of the patients. However, the types 3 and 6 are increasing year by year, which shows that the HCV genotypes in the injection drug users are transmitting to the general population. The status of HCV genotype coexistence and the increase of 6a may affect the overall HCV genotype distribution pattern in China. Moreover, according to report, the diversification of HCV transmission modes, such as sexual transmission is gradually becoming the main route of transmission in the central region of the country [41]. According to the sentinel surveillance, the HIV transmission route in Guangdong had shifted from IDUs to sexual transmission since 2008 [42]. To be much more concerned, Guangdong may become an important source of HCV epidemic, causing significant changes of HCV genotypes or subtypes via a variety of modes of transmission. The changing status of HCV subtypes will make a direct impact on HCV diagnosis, prognosis, treatment of hepatic diseases and control and prevention of HCV. 

## Supporting Information

Table S1 **Multivariate analysis of factors associated with HCV infection.** A two-side *p* < 0.05 was considered to be significant. Univariate analysis was used to calculate the crude and adjusted odds ratios (OR) and their 95% confidence intervals (CI). Multivariate analysis was performed with logistic regression using HCV infection as dependent variable and risk factors screened by univariate analysis as independent variables.
(DOC)
