# Correction: Molecular Epidemiologic Characterization of a Clustering HCV Infection Caused by Inappropriate Medical Care in Heyuan City of Guangdong, China

**DOI:** 10.1371/annotation/19a005e0-dda8-4883-b48c-adfeb60796e0

**Published:** 2014-01-07

**Authors:** Yi-Qun Kuang, Jin Yan, Yan Li, Xuhe Huang, Ye Wang, Guolong Yu, Xinge Yan, Ping Lin, Bing Qin, Peng Lin

This article was republished on Jan 3rd, 2014 due to figures missing in the PDF. Please download the new PDF to view the figures.

