# Supplementary material for: Molecular Epidemiologic Characterization of a Clustering HCV Infection Caused by Inappropriate Medical Care in Heyuan City of Guangdong, China
Source: PLoS One. 2013 Dec 3;8(12):e82304. doi: 10.1371/journal.pone.0082304 (PMC3857772; doi:10.1371/journal.pone.0082304)
Supplement: Table S1 — Multivariate analysis of factors associated with HCV infection. A two-side p < 0.05 was considered to be significant. Univariate analysis was used to calculate the crude and adjusted odds ratios (OR) and their 95% confidence intervals (CI). Multivariate analysis was performed with logistic regression using HCV infection as dependent variable and risk factors screened by univariate analysis as independent variables. (DOC) [file pone.0082304.s001.doc]

**Table S1.** Multivariate analysis of factors associated with HCV infection.

|  | Numbers | | exposure% | |  |  |
| --- | --- | --- | --- | --- | --- | --- |
|  | Cases | Controls |  |  |  |  |
| Factors | n = 184 | n = 172 | Cases | Controls | *p* value | OR (95% *CI*) |
| Blood transfusion | 12 | 3 | 6.52 | 1.74 | 0.721 | 1.4 (0.2-8.9) |
| Operation | 41 | 20 | 22.28 | 11.63 | 0.452 | 1.4 (0.6-3.3) |
| Intravenous transfusion | 129 | 99 | 70.11 | 57.56 | 0.154 | 1.6 (0.8-3.0) |
| Intravenous Infusion | 163 | 44 | 88.59 | 25.58 | <0.001 | 20.4 (10.7-38.8) |
| Oral Check | 81 | 46 | 44.02 | 26.74 | 0.055 | 1.9 (1.0-3.5) |
| Endoscope Exposure | 23 | 7 | 12.50 | 4.07 | 0.016 | 4.2 (1.3-13.8) |
| Bioproducts Infusion | 49 | 25 | 26.63 | 14.53 | 0.175 | 1.7 (0.8-3.6) |
| Acupuncture | 18 | 7 | 9.78 | 4.07 | 0.149 | 2.6 (0.7-9.9) |
| Partner/Parents/Children HCV^+^ | 83 | 18 | 45.11 | 10.47 | <0.001 | 4.6 (2.3-9.4) |

Abbreviations: OR, odds ratio; CI, confidence interval
